# Beyond the Role of Dietary Protein and Amino Acids in the Prevention of Diet-Induced Obesity

**DOI:** 10.3390/ijms15011374

**Published:** 2014-01-20

**Authors:** Klaus J. Petzke, Anne Freudenberg, Susanne Klaus

**Affiliations:** German Institute of Human Nutrition in Potsdam-Rehbruecke (DIfE), Arthur-Scheunert-Allee 114-116, Nuthetal 14558, Germany; E-Mails: anne_freudenberg@gmx.de (A.F.); klaus@dife.de (S.K.)

**Keywords:** high-protein diet, leucine supplementation, energy intake, water intake, metabolic syndrome, diet-induced obesity

## Abstract

High-protein diets have been shown to prevent the development of diet-induced obesity and can improve associated metabolic disorders in mice. Dietary leucine supplementation can partially mimic this effect. However, the molecular mechanisms triggering these preventive effects remain to be satisfactorily explained. Here we review studies showing a connection between high protein or total amino nitrogen intake and obligatory water intake. High amino nitrogen intake may possibly lower lipid storage, and prevent insulin resistance. Suggestions are made for further systematical studies to explore the relationship between water consumption, satiety, and energy expenditure. Moreover, these examinations should better distinguish between leucine-specific and unspecific effects. Research in this field can provide important information to justify dietary recommendations and strategies in promoting long-term weight loss and may help to reduce health problems associated with the comorbidities of obesity.

## 1. Introduction

**Figure 1.**
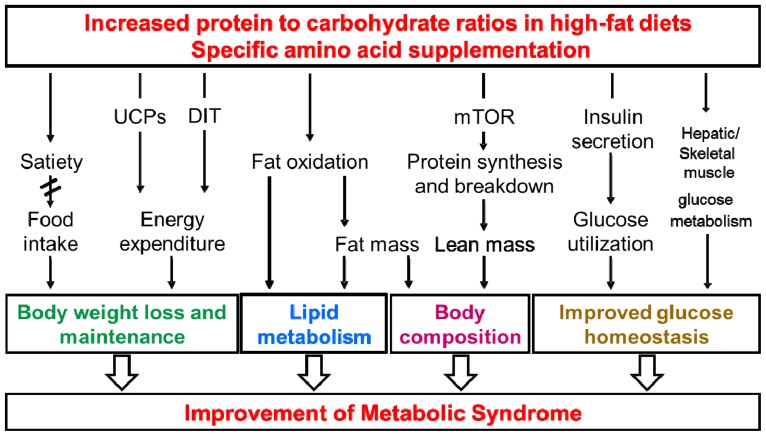
Simplified scheme of possible mechanisms of beneficial effects of dietary protein on the metabolic syndrome. UCP, uncoupling protein; DIT, diet-induced thermogenesis; mTOR, mammalian target of rapamycin.

The epidemic dimension of obesity prevalence and related disorders represents an increasing problem in both developed and developing countries [1]. Therefore, it is a challenge to find effective treatments and preventive strategies to reduce the extent of overweight. One of the dietary approaches is the consumption of high-protein diets that can promote weight loss and weight maintenance in animals and humans. Further, high-protein diets could improve glucose homeostasis, increase energy expenditure (EE), may lower blood lipids, can reduce blood pressure, and could preserve lean body mass [2–7]. Similar effects have been demonstrated in mice when the protein to carbohydrate ratio in a high-fat diet was increased [8–11]. In addition, dietary supplementation of functionally different single amino acids including arginine, glutamine, glycine, and leucine may improve glucose and lipid metabolism [12–18]. However, the underlying mechanisms of these beneficial effects are not fully understood and are areas of controversy (summarized in Figure 1).

Our own studies [8,19,20] were aimed to investigate the effects of long-term high-whey protein diets containing either a normal or high-fat content on body weight (BW) regulation and EE to clarify the potential role of high-quality protein consumption in the prevention of metabolic syndrome associated traits in mice. It was suggested that the metabolic improvements related to high-protein intake could be mediated by specific amino acids such as the nutritionally indispensable branched-chain amino acid leucine. This suggestion results from several studies that have shown obvious advantageous effects of leucine supplementations on obesity development and/or glucose homeostasis using different rodent models of obesity and diabetes mellitus [18,21–24]. In principle, leucine may have specific functions related to its action on the mTOR pathway in hypothalamus and muscle which could affect food intake and energy homeostasis as well as skeletal muscle protein synthesis or breakdown [25–30]. However, so far it is not confirmed that a long-term leucine supplementation results in a sustained stimulation of protein synthesis and prevention of protein loss [31]. We investigated whether leucine might be responsible for the high-protein mediated effects during short-term and long-term interventions. To this end, we compared high protein (HP) whey diets (50% *w*/*w*, HP) with either adequate protein (AP) control (10% *w*/*w*, AP) or AP diets supplemented with leucine (AP + L) diets matching the leucine content of HP. Moreover, we investigated the effects of alanine supplementation using an additional control (AP + A). The AP + A diet was equimolar in alanine concentration compared with leucine of the AP + L diet [8,19]. This was done to distinguish leucine specific effects from unspecific amino acid effects. All other studies we are aware of did not include controls of alternative amino acid supplementations in comparison to the leucine supplementations.

## 2. Amino Acids and Insulin Signaling

It was recognized that mTOR can sense the availability of amino acids which regulate insulin sensitivity and may cause an inhibition of glucose uptake via phosphorylation of downstream factors of insulin signaling cascade by S6 kinase 1 (S6K1) [32,33]. However, the function of leucine in the modulation of glucose tolerance and insulin resistance remains an area of controversy. Whereas an improvement of glucose tolerance and insulin signaling was demonstrated, possibly due to dietary leucine in rodents [18,34,35], animal and human studies have shown that leucine impairs glucose tolerance [36] and can promote insulin resistance [32,33,37–40]. Additional studies showed no effects of leucine on glucose and insulin homeostasis [41–44]. These controversies may be generally due to dose effects, different experimental conditions such as differences in administration forms, and the animal models used [45]. In principle, modulations of glucose homeostasis by dietary leucine can involve different direct and indirect mechanisms such as stimulation of insulin and glucagon secretion, changes of hepatic glucose metabolism, impairment of insulin signaling, and glucose uptake in skeletal muscle [34,36,46]. Amino acids and insulin were suggested to synergistically activate S6K1 and to induce phosphorylation of insulin receptor substrate 1 (IRS-1). This activation may cause insulin resistance by direct inhibition of skeletal muscle glucose transport [32,47,48]. Interestingly, leucine was identified so far as the only amino acid directly interacting with the insulin pathway as a signaling molecule [49]. In contrast, long-term supplementation of leucine did not result in a sustained activation of the mTOR pathway. The insulin-stimulated glucose uptake into skeletal muscle was improved without changes in overall glucose tolerance in old rats [44]. Furthermore, it was suggested that glucose homeostasis can be maintained by an increased recycling of glucose via the glucose-alanine-cycle stimulated by dietary leucine [50,51]. Additionally, leucine and its metabolites were shown to directly activate Sirt1 (a NAD^+^-dependent deacetylase implicated in metabolic regulation) and Sirt1-dependent pathways of fat oxidation and insulin signaling in a cell-free system [52,53]. Sirt1 activation in liver, brown adipose tissue, and gastrocnemius muscle was shown after leucine supplementation to drinking water, which may contribute to the prevention of metabolic dysfunctions and insulin resistance in high-fat diet fed mice [54]. Taken together, these data demonstrate that the role of leucine in glucose homeostasis and insulin resistance is not completely understood. Moreover, it is not clear whether these effects are leucine-specific or not. Clarification of a causal relationship could contribute to an explanation of the observed diverse effects of high-protein diets on insulin resistance and diabetes risk in animal and human studies [40,55–57]. Therefore, further research is warranted to determine the mechanisms by which dietary interventions such as high-protein intake can improve metabolic health [3,58].

## 3. Body Weight and Body Composition

**Figure 2.**
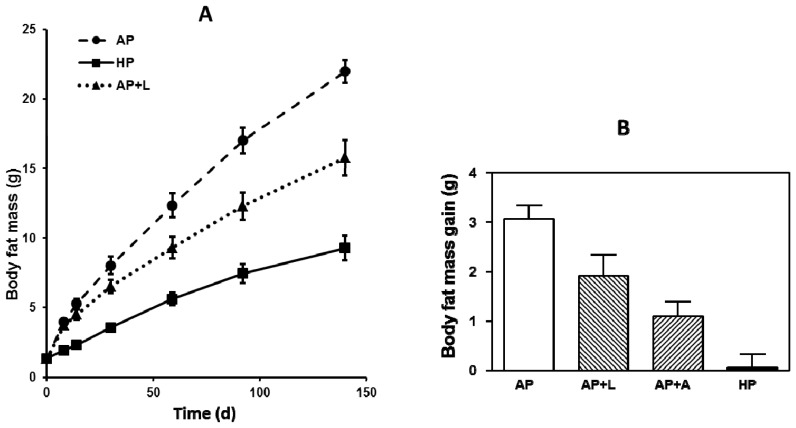
(**A**) Long term body fat accumulation. Ten weeks old male C57BL/6 mice were fed for 20 weeks *ad libitum* with experimental semisynthetic high-fat diets (20% *w*/*w* of fat) containing different protein and leucine concentrations. AP, adequate protein (10% *w*/*w* of whey protein); HP, high-protein (50% *w*/*w* of whey protein); AP + L, AP supplemented with l-leucine corresponding to HP (+6% l-leucine) [8]; (**B**) Short term body fat mass gain. Ten weeks old male C57BL/6 mice were fed for 7 days *ad libitum* with experimental semisynthetic high-fat diets (20% *w*/*w* of fat) containing different protein and leucine and alanine concentrations. AP, adequate protein (10% *w*/*w* of whey protein); HP, high-protein (50% *w*/*w* of whey protein); AP + L, AP supplemented with l-leucine corresponding to HP (+6% *w*/*w* l-leucine, 0.572 mole); AP + A, AP supplemented with equimolar l-alanine (+4.5% *w*/*w* l-alanine, 0.572 mole). Data are means ± SEM, *n* = 9–10 [19].

We have shown that a high-fat diet-induced body fat accumulation can be reduced by simultaneous high protein and leucine administration, both in the short and long term (Figure 2). Interestingly, equimolar supplementation with alanine decreased body fat mass gain in a short-term experiment similarly as leucine compared with AP-fed mice, although the metabolic functions of both amino acids are quite different [8,19].

Chronic HP exposure also increased lean body mass whereas weights of *m. quadriceps* were higher in both HP and AP + L long-term experiments. This may be explained through a positive skeletal muscle protein balance. Indeed, we suspected increased rates of skeletal muscle protein synthesis because of higher incorporation of ^15^*N*-lysine after long term HP and AP + L exposures [8]. In contrast, other studies did not find an increase in skeletal muscle mass by long-term supplementation of leucine in humans and rats [31,44]. Branched-chain amino acids and particularly leucine are suggested to regulate muscle protein synthesis by activation of the mTOR pathway and thereby to stimulate protein synthesis on the translational level [27,30,59]. However, we did not find any evidence of activation of mTOR or its downstream targets (such as eIF4E-binding protein 1 (4E-BP1) and the ribosomal S6 protein (rS6-P) in skeletal muscle in our experiments ([8,19]). This is in contrast to other publications emphasizing the role of leucine as a potent activator of the mTOR pathway, which could be due to species differences or different experimental set-ups [27,30,60,61].

Consequently, we suggest that alternatively, other mechanism may contribute to the long-term regulation of skeletal muscle mass by dietary protein and leucine. In this context, a depression of protein degradation following leucine supplementation was reported [62–65]. Inhibitions of the ubiquitin-proteasome [66,67] or the autophagy-lysosome systems [68] by leucine were suggested to trigger suppressions of muscle protein breakdown. However, anti-proteolytic functions of leucine are less documented than effects on muscle protein synthesis, which deserves further investigation.

## 4. Energy and Water Intake

**Figure 3.**
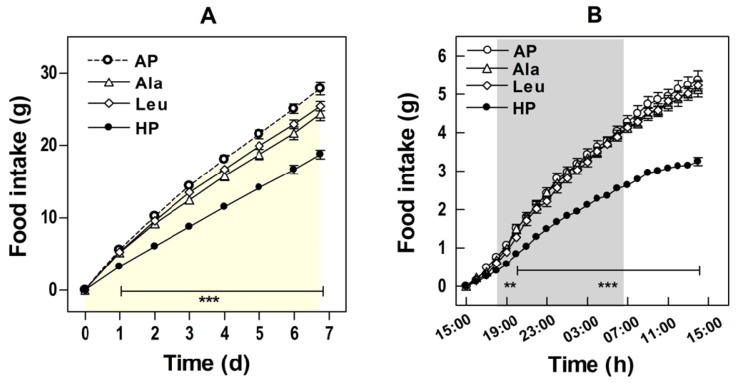
Food intake. Ten weeks old male C57BL/6 mice were fed for seven days *ad libitum* with experimental semisynthetic high-fat diets (20% *w*/*w* of fat) containing different protein and leucine and alanine concentrations. AP, adequate protein (10% *w*/*w* of whey protein); HP, high-protein (50% *w*/*w* of whey protein); Leu, AP supplemented with l-leucine corresponding to HP (+6% *w/w* l-leucine, 0.572 mole); Ala, AP supplemented with equimolar l-alanine (+4.5% *w*/*w* l-alanine, 0.572 mole). (**A**) Cumulative food intake during the whole feeding trial; (**B**) Food intake on the first day of dietary intervention. Values are means ± SEM. The shaded area refers to night (light off) period. Asterisks indicate significant differences from AP (** *p* < 0.01, *** *p* < 0.0001) [19].

A higher satiating effect of dietary protein was assumed to be the main cause of high-protein diet induced weight loss [7,69]. This is in line with human investigations showing no changes in BW following isoenergetic intakes of high-protein and high-carbohydrate diets [70–73]. Hence, it was assumed that the reduced energy intake and not the macronutrient composition was likely to be crucial for loss of BW following hypo-caloric diets [71,72,74,75]. A higher satiety after high-protein diets might also result in improved weight maintenance because of better acceptance and compliance as compared with low-protein meals [76]. Our experiments with mice were performed under *ad libitum* conditions and have shown a significantly reduced food intake of HP and AP + L as compared with AP exposed controls with a strong correlation of energy intake with BW gain [8,19]. High-protein diets have also been shown to prevent the initial hyperphagia induced by high-fat feeding and thus can delay the development of obesity [9]. This was confirmed in a short-term study [19] showing that the intake of a high-fat diet was acutely affected by HP supplementation with significant effects apparent as early as five hours after the dietary switch (Figure 3).

Interestingly, the supplementation with leucine as well as with alanine led to a reduction in energy intake. However, the extent of this reduction was less compared with HP exposure. Increases in circulating leucine concentrations have been suggested to play a role in hypothalamic regulation of food intake by acting as satiety signal [25,26,28]. As expected, in our study plasma leucine was only increased in the HP and AP + L groups and not in AP + A group [19]. Therefore, changes in circulating leucine concentrations cannot explain the effect on satiety and fat accumulation alone. Furthermore, because alanine supplementation did not alter plasma amino acid levels, it seems unlikely that changes in concentrations of circulating amino acids contribute to the satiety effect we observed [19].

Summarizing, although we could show that food or energy intake is affected by dietary protein and amino acids it seems unlikely that the increase in satiety depends on hypothalamic regulation by leucine. This contrasts with conclusions associating leucine with neural mechanisms [29,77]. However, it is necessary to clearly distinguish the experimental conditions among different studies such as the energy consumed from fat. Furthermore, it became evident that energy intake is responsible to a high degree for changes in BW and body fat in mice exposed to adipogenic high-fat diets.

**Figure 4.**
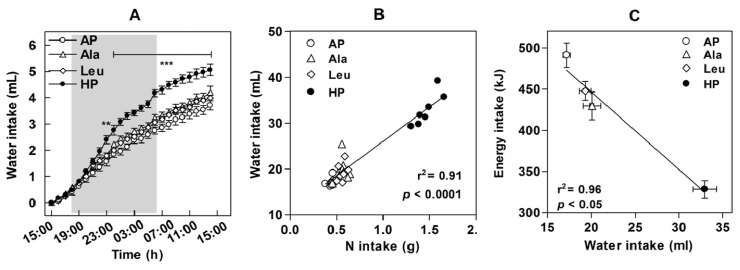
Water intake. Ten weeks old male C57BL/6 mice were fed for seven days *ad libitum* with experimental semisynthetic high-fat diets (20% *w*/*w* of fat) containing different protein and leucine and alanine concentrations. AP, adequate protein (10% *w*/*w* of whey protein); HP, high-protein (50% *w*/*w* of whey protein); Leu, AP supplemented with l-leucine corresponding to HP (+6% *w*/*w* l-leucine, 0.572 mole); Ala, AP supplemented with equimolar l-alanine (+4.5% *w*/*w* l-alanine, 0.572 mole). (**A**) Water intake on the first day of dietary intervention; (**B**) Correlations of water intake with nitrogen intake; (**C**) Correlations of water intake with energy intake. Values in (**B**) and (**C**) are means ± SEM (** *p* < 0.01, *** *p* < 0.0001) [19].

Additionally, we also measured water consumption of mice exposed to the experimental diets during a short-term study [19] (Figure 4) because we suspected a role of water consumption on satiety and BW (for review see: [78]). Human studies demonstrated that drinking of water can increase satiety, reduce energy intake, and induce weight loss [79–81]. We observed an almost doubled water intake of mice fed the HP diet as compared to the AP group [19]. Although the increase in water consumption of the leucine or alanine supplemented groups did not reach statistical significance, overall water intake strongly correlated with dietary amino nitrogen intake and negatively correlated with energy intake in all groups (Figure 4). Similar increases in water intake were observed in high-fat diet fed mice receiving whey protein supplemented in drinking water resulting in 78% greater levels of protein consumption compared to unsupplemented controls [10]. It is possible that this effect is related to urea production, which increases in parallel with the level of ingested protein or amino nitrogen to handle the excess of amino acids consumed [82,83].

## 5. Energy Expenditure (EE)

Additional effects contributing to the leaner phenotype of high-protein diet exposed mice might be an increase in diet-induced thermogenesis (for review see: [7,69]). We could show that both HP diets and leucine supplementation can increase weight specific resting EE to the same extent [8]. This supported the suggested role of HP diets and of leucine in increasing EE [7,51]. EE is predominantly a function of fat free body mass [84–86]. Therefore, our observations are in line with higher *m. quadriceps* weights of mice following long-term HP or AP + L exposures [8].

Interestingly, differences in water consumption [19] might also have had an impact on EE. It was reported that metabolic rate can increase by about 30% in healthy normal-weight subjects following drinking of 500 mL of water [87]. EE was also enhanced following water intake in overweight and obese subjects [88]. In general, influences of water intake on osmolality, thermogenesis, and on metabolic organ functions have been discussed [88]. However, a connection between water intake induced by dietary protein or amino acids and changes in EE and other metabolic functions is so far only hypothetical and needs to be investigated more systematically.

Previously we also suggested that higher rates of EE might be caused by increased uncoupling protein (UCP) expression following high-protein and leucine intake [18,89]. This suggestion resulted from studies measuring mRNA expressions in rats after long- term or short-term exposures with high-protein diets [89,90]. However, we could not detect any significant changes in the protein expression of UCP homologues in mice in either short-term or long-term experiments which argues against a participation of UCPs in high-protein or leucine-mediated effects on EE [8]. Overall, the data show that diets high in protein or leucine concentration can affect EE. But the extent of its contribution to BW reduction and the mechanisms remains to be elucidated.

## 6. Lipid Metabolism and NAFLD

**Figure 5.**
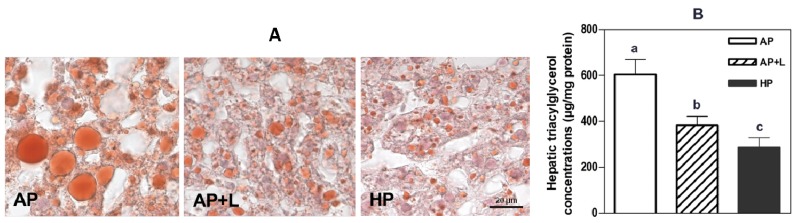
Oil red O staining of livers (**A**) and hepatic triacylglycerol concentrations (**B**). Ten weeks old male C57BL/6 mice were fed for 20 weeks *ad libitum* with experimental semisynthetic high-fat diets (20% *w*/*w* of fat) containing different protein and leucine concentrations. AP, adequate protein (10% *w*/*w* of whey protein); HP, high-protein (50% w/w of whey protein); AP + L, AP supplemented with l-leucine corresponding to HP (+6% l-leucine). Data are means ± SEM, *n* = 9–10, “a, b, c” indicates that “*p* < 0.05” [8].

We could show that long-term HP exposure prevents high-fat diet induced fat accumulation in liver which was mimicked to a considerable extent by leucine supplementation (Figure 5, [8]). Significant lower liver triacylglycerol concentrations were also observed in mice after only one week of dietary exposure with the HP diet. In the short term, leucine or alanine supplementations did not (yet) show such significant effects on liver fat [19]. Although, the gain in fat mass was highly correlated with energy intake in these studies, only about 77% of body fat increase could be explained by energy intake. Fewer hepatic fat contents were also found in mice fed with high whey protein containing high-fat diets compared to controls without significant differences in overall energy consumption. [10] Lower feeding efficiency and modifications of energy expenditure and oxygen consumption after high-protein diet exposures can indicate mechanisms resulting in negative energy and fat balance [10,89,90].

In general, liver represents a key organ for maintenance of energy homeostasis. Therefore it is very likely that disturbances in hepatic metabolism are linked to events contributing to the metabolic syndrome [91,92]. Several processes leading to a fatty liver phenotype are discussed. These include higher rates of hepatic *de novo* lipogenesis, reduced lipid oxidation, increased uptake of fatty acids, and defective discharges of lipids. Our gene expression data using adipogenic diets clearly indicated that lower hepatic triacylglycerol concentrations in long-term HP fed mice [8] can be linked to reduced rates of hepatic *de novo* lipogenesis. In particular, lower mRNA levels of acetyl-CoA-carboxylase alpha (ACCα) were observed in HP diet exposed mice compared with AP controls. However, hepatic mRNA levels of ACCα in leucine supplemented AP + L fed mice were not different from AP controls. This suggests that the HP diet effects on liver lipid metabolism could be rather due to the lower dietary carbohydrate intake with the HP diet and that leucine did not exhibit specific effects [8]. Similarly, mRNA expression of ACCα was also decreased in livers of mice exposed to HP diet for one week while supplementations with alanine or leucine did not reduce ACCα expression significantly (Table 1).

Furthermore, it was shown that an increased fatty acid uptake due to higher expression levels of lipid transport proteins such as fatty acid translocase (CD36) and liver-type fatty acid binding protein (l-FABP) can be more important for deposition of fat in the liver than changes in *de novo* lipogenesis or fat oxidation [93]. Consequently, the reduced hepatic lipid accumulation in HP and AP + L exposed mice could result from decreased fatty acid uptake due to lower protein levels of CD36. This was observed after long-term feeding of HP as well as of AP + L [8]. In contrast, CD36 protein as well as its mRNA level was not modified in livers of mice fed HP, AP + A, or AP + L diets for only one week (Table 1). Hepatic triacylglycerol concentrations did not differ significantly between amino acid supplemented groups and AP fed controls after one week. However, we cannot exclude an influence of dietary amino acids on hepatic fatty acid uptake because l-FABP mRNA was significantly reduced in all intervention groups compared to AP (Table 1). Interestingly, HP feeding seems to stimulate basal lipolysis in white fat as indicated by increased gene expression of adipose triacylglycerol lipase (ATGL) after one and 20 weeks of high-fat feeding ([8]; Table 1).

However, this was not observed in the AP + L and AP + A groups (Table 1), which again might be due to the lower carbohydrate intake of the HP group. Therefore, high-protein exposure and leucine supplementation were able to prevent hepatosteatosis in mice possibly due to different mechanisms. The HP effects on liver lipogenesis and adipose tissue lipolysis could primarily be due to a lower carbohydrate intake while the effects on hepatic fatty acid uptake could also be influenced by single amino acid supplementations.

Alternative mechanisms in prevention of hepatosteatosis are not well-studied so far in particular in the context of the advantageous effects of high-protein diets. Therefore, more detailed studies of alterations in expression and regulation of key enzymes and of transcriptional co-factors controlling hepatic lipid and energy metabolism are required.

## 7. Specificity of Observed Leucine Effects

Obviously, some of the beneficial effects of high-protein diets in the prevention of disadvantageous effects of adipogenic diets can be mimicked by dietary leucine supplementation [8]. This supports the significance of leucine as a potential nutritional treatment against obesity, insulin resistance, and NAFLD. However, experimental studies did not include additional control groups comparing the effects of alternative amino acids with those of leucine. Therefore, we have compared an additional diet group as control by supplementing alanine in a short-term intervention in mice [19]. Surprisingly, the equimolar substitution of leucine by alanine resulted in comparable effects of both supplementations on BW and body fat mass, food intake, water consumption, and hepatic triacylglycerols [19]. This implies that some or even most changes in energy and substrate metabolism induced by high-protein diets are not specifically related to leucine but seem to be rather a result of increased amino nitrogen consumption. Further long-term experiments are needed to investigate the specificity of leucine effects on metabolic disorders induced by adipogenic diets. It is striking that none of the published studies investigating the effects of leucine supplementation on metabolic syndrome related traits included a control group supplemented with another amino acid or another source of amino nitrogen [18,24,44]. We suggest that this is essential in order to delineate which metabolic effects and mechanisms are amino acid-specific or a result of higher amino nitrogen consumption and its metabolic consequences.

## 8. Metabolic Consequences of Increased Water Intake in Response to High-Protein Diets

The observed association between increased water intake and lower energy intake led us to suspect that higher amino nitrogen intake resulting in raised urea production could cause the stimulation of obligatory water consumption [19]. It was shown in pigs that the excretion of higher amounts of urea resulted in the need for a higher volume of urine [94]. On the other hand there are some results indicating that higher water consumption may have advantageous metabolic consequences in obesity and diabetes in children and adults [95,96]. Firstly, epidemiological data suggest that water drinkers lower their energy intake. Further, pre-meal water consumption reduces energy intake and daily water intake may facilitate long-term weight loss in older adults [78,97]. It was also observed that a promotion of water drinking can effectively reduce weight gain among school children [98]. Sympathetic activation [99] and increases in metabolic rate [87] were detected following water intake in humans. Secondly, animal studies showed that higher protein concentrations in experimental diets significantly enhanced water intake in mice [100], rats [101–103], and pigs [94] accompanied by higher urine volumes. Interestingly, mice fed high-protein diets exhibited higher liver water contents [91]. Obviously, water drinking can produce intracellular hydration to prevent a hyperosmotic state and alterations in cell volume and hydration are known to regulate metabolism of proteins, glucose, and amino acids, and modify the expression of a wide variety of genes. The increase in amino acid concentrations following their uptake is recognized as one of the signals contributing to liver cell hydration [104,105]. Furthermore, it was shown in a rat study that the inhibition of angiotensin converting enzyme reduced body fat mass and plasma leptin while doubling the intake of water [106]. This suggests that either inhibition of the renin-angiotensin system unblocks fat metabolism or that increased water intake *per se* can regulate fat metabolism [107]. Moreover, cell dehydration was shown to inactivate mTOR signaling and to decrease insulin-induced glucose uptake [108]. Furthermore, a deficiency of TRPV4 (a transient receptor potential channel regulating cell volume by Ca^2+^ channeling which is activated by osmolarity) was shown to increase muscle oxidative capacity and resistance to diet-induced obesity in mice [109]. Nevertheless, a transient hypo-osmolarity was shown to increase whole-body lipid turnover in humans [110,111]. Thus, cell hydration should have opposite effects [107]. Consequently, more systematical investigations are needed to delineate the mechanisms of increases in water intake following high-protein diets and its consequences on metabolic syndrome related traits for a better explanation of the efficiency of high-protein diets.

## 9. Conclusions

We have shown that high-protein diets are able to prevent the development of diet-induced obesity and that they improve associated metabolic disorders in mice. These effects were mimicked by leucine supplementation although less pronounced [8]. Further, all metabolic effects of leucine supplementation seem to be a consequence of the concomitant increase in dietary amino nitrogen at least in the short-term [19]. However, the mechanisms remain to be satisfactorily explained. Long-term intervention studies comparing leucine supplementation with equimolar supplementation of other amino acids should be performed to distinguish between leucine-specific and unspecific effects. Furthermore, the physiological conditions in which leucine can activate mTOR and therefore affect muscle protein synthesis, glucose homeostasis, and food intake have to be elucidated. It seems reasonable to investigate skeletal muscle protein turnover with alternative methods to clarify the underlying mechanisms by which dietary protein and leucine affect body composition. Nevertheless, water intake was found to correlate with amino nitrogen consumption in different experimental groups. Therefore, water consumption was suggested to be related to satiety and EE [19] which should be studied more systematically. Studies in this field can increase our knowledge about the mechanisms of obesity prevention and metabolic syndrome related traits.

## Figures and Tables

**Table 1.** Relative gene expression levels of liver and white adipose tissue proteins from ten weeks old male C57BL/6 mice fed different diets with an adequate (AP), a high (HP) or an adequate protein concentration supplemented with l-alanine (AP + A) or l-leucine (AP + L) for one week [19] 1,2.

	AP	AP + A	AP + L	HP	*p* <
**Liver**					
ACCα	1.00 ± 0.09 ^a^	0.78 ± 0.12 ^a^	0.67 ± 0.09 ^a^	0.35 ± 0.03 ^b^	0.01
CD36	1.00 ± 0.08	0.73 ± 0.10	0.99 ± 0.10	1.04 ± 0.16	NS
FAS	1.00 ± 0.26 ^a^	0.55 ± 0.10 ^b^	0.37 ± 0.05 ^b^	0.18 ± 0.03 ^b^	0.05
L-FABP	1.00 ± 0.14 ^a^	0.77 ± 0.05 ^b^	0.59 ± 0.05 ^b,c^	0.48 ± 0.03 ^c^	0.05
**Epididymal white fat**					
ATGL	1.00 ± 0.07 ^a^	1.28 ± 0.15 ^a^	1.38 ± 0.13 ^a^	1.86 ± 0.25 ^b^	0.01
HSL	1.00 ± 0.05	1.10 ± 0.10	0.89 ± 0.15	1.21 ± 0.08	NS

1 Values are means ± S.E.M., *n* = 6–8. Within a row, values without a common superscript differ significantly; NS, not significant;

2 ACCα, acetyl CoA carboxylase; CD36, fatty acid translocase; FAS, fatty acid synthase; L-FABP, liver-type fatty acid binding protein; ATGL, adipose triacylglycerol lipase; HSL, hormone sensitive lipase.
